# 

**Published:** 1874-07

**Authors:** 


### AMERICAN DENTAL ASSOCIATION.

The fourteenth annual meeting of the American Dental Association will be held in the city of Detroit, on Tuesday, the 4th of August, and continue four days. Judging from the arrangements under way and already made, we are assured that the meeting will be a large and profitable one, and we trust it will draw together the large body of the energetic, progressive working men of the profession.

### ATTENTION.

### AMERICAN DENTAL ASSOCIATION.

The fourteenth annual meeting of the American Dental Association will be held in the city of Detroit, on Tuesday, the 4th of August, and continue four days. Judging from the arrangements under way and already made, we are assured that the meeting will be a large and profitable one, and we trust it will draw together the large body of the energetic, progressive working men of the profession.

Jt is very desirable that they should come together with a firm resolve to labor for the best interests of the profession, and with a preparation that shall enable them to do this most successfully. Let not anything be done that shall call forth such criticism as has sometimes been made upon the doings of this body. Of course, everything is liable to criticism, but let every thing be done so that just criticism would make it appear better than worse.

The following list of standing committees will give an idea of the subjects to be presented:

Physiology, M. S. Dean, E. A. Bogue, A. W. Harlan.

Pathology and Surgery, H. Judd, H. S. Chase, C. C. Knowles.

Histology and Microscopy, T. B. Hitchcock, C. E. Latimer, J. Taft.

Chemistry, H. A. Smith, C. R. Butler, W. T. Wallace.

Therapeutics, T. C. Stellwagen, L. Jack, J. McManus.

Operative Dentistry, L. D. Shepard, G. C. Daboll, G. L. Field.

Mechanical Dentistry, S. B. Brown. J. F. Canine, E. D. Swain

Dental Education, A. F. McLain, G. W. Keely, E. J. Waye.

Dental Literature, W. H. Eames, Chas. Buffett, P. G. C. Hunt.

Dental Etiology, J. H. McQuillen, M. H. Webb, James Johnstone.

Prize Essay, W. H. Goddard, Isiah Forbes, G. F. S. Wright.

---

### SOUTHERN DENTAL ASSOCIATION.

We trust the following announcement and programme will interest the profession sufficiently to draw together a large assemblage of its members. We have had the priviledge of attending this Association but once and from our experience that time, we can most sincerely urge all who possibly can to attend. The probality is that this will be one of the largest and most interesting meetings of this boby, ever held. Then let all go who can and every one go prepared to contribute.

ED.

The sixth annual meeting of the Southern Dental Association will be held in Polytechnic Hall, St. Louis, Mo., commencing at 10 o'clock a. m. Tuesday, July, 28, 1874. Dr. Isaiah Forbes of St. Louis will deliver the address of welcome.

The following subjects are suggested for essays and discussions:—

1 Teething, and treatment of diciduous teeth.

2 Irregularities of permanent teeth; cause and treatment.

3 Best material for filling thc roots of teeth.

4 Best materials for crown fillings, and conditions indicating their use.

5 Artificial dentistry; what bases are best.

6 Hasty operations *vs.* common honesty.

7 Contour fillings; their merits and demerits.

8 Dental Education.

9 Legislation regarding dentistry.

10 Best method of devitalizing teeth, and of saving dead teeth; indicating conditions.

11 Dental histology and microscopy.

12 Dental phyiology and anatomy.

13 Dental chemistry and metallurgy.

14 Primal cause of decay and loss of teeth.

15 Rubber and other diseases, from malpractice.

### AMERICAN DENTAL CONVENTION.

We take pleasure in directing attention to the following notice. This is one of the popular institutions of the profession, one that ought to call together a large body annually. In it entire freedom is given to all to participate in the discussions and proceedings, the largest latitude is given in the presentation and treatment of subjects.

It is twenty years since its organization, and during that time it has accomplished much. It has to some extent, been the means of increasing the stock of knowledge of many and of indirectly aiding others by stimulating them to greater effort and higher purpose, and the professional social element, is a very important one. The more we come together and the better we know each other, the better it is for all concerned, and the American Dental Convention has had a wide-reaching influence in this respect, then let all who can possibly do so be present. But to the circular.

Editor Dental Register:—

*Dear Sir.* Permit me through the medium of your influential journal to call the attention of its readers to the meeting of the American Dental Convention, which holds its twentieth annual session at Saratoga, on the eleventh of August.

This organization has done much towards the elevation of our profession. Established on democratic principles, opening its doors to all reputable practicing dentists.

If a tree is known and appreciated by its fruits surely this convention should occupy a prominent position in the Dental World, numbering as it does among its members many of the brightest intellects in our ranks.

The friends of the convention anticipate a large attendance at its coming session. Dr. T. W. Evans, of Paris, is expected to deliver the opening address and many of the most prominent and influential members of the profeesion will be in attendance, read papers and participate in the discussions etc.

The transactions for the three past years are in press and will be ready for distribution at or before the meeting.

A cordial invitation is extended to every practicing dentist who feels interested in and desires the advancement of Dental Science and knowledge, to be present and contribute his mite towards that end and assist in pushing on the wheels of progress, and thus keep pace with the times and other specialties of Medicine and Surgery.

Let all who love our profession and its advancement be sure to be there. J. G. A.

### THE OHIO COLLEGE OF DENTAL SURGERY.

This Institution has just issued its twenty-ninth annual announcement. There have been some changes in the Faculty since last session.

The Chair of Physiology was vacated before the close of last session, by the resignation of Prof. Rives, and was filled by the appointment of Dr. J. L. Cilley, who has been a teacher in the Miami Medical College of this city for several years.

The Chair of Anatomy was vacated at the close of the last session by Prof. Kearns, and Prof. Wm. Clendenin was appointed its occupant.

The doctor has been for several years Professor of Anatomy in the Miami Medieal College, and has established a high réputation as a teacher. He was also health officer of this city for a number of years. Prof. Hunter resigned the chair of Mechanical Dentistry, and Dr. Wm. Van Antwerp of Mt. Sterling, Kentucky, a graduate of this Institution, was appointed to fill the position. We are well assured that everything possible will be done to make the approaching session a profitable and efficient one. The Faculty propose to arrange the order of exercises so that there shall not only, not be any unoccupied time, but that every moment shall be employed to the best advantage for the student. There are some qualities that ought to be possessed by every dental student.

1. He ought to have some natural ability for that which he is to undertake.

2. He should have a love for his work.

3. He should have a proper estimate of the work upon which he is entering.

4. He should have a just appreciation of its character and importance.

5. He should have a fixed purpose of accomplishing fully, and in the best manner possible the work he attempts.

6. He should set his mark high and never stop short of it.

7. He ought to have a good literary and scientific education, the more of each the better.

8. He should be honest.

9. He should be industrious and persevering.

10. He should have the elements of true manhood in large measure.

11. His habits should be good.

12. The student before entering the college should have some preliminary instruction in dentistry.

The Faculty would doubtless be glad to fill up the class for the coming session with students possessing these qaulifications.

### AMERICAN ACADEMY OF DENTAL SCIENCE.

The seventh annual meeting of the American Academy of Dental Science will be held in Boston, on Monday, September 28th, 1874, at 10 o'clock a. m.

The annual address will be delivered by Dr. W. W. Allport, of Chicago. E. N. Harris, Cor. Secretary.

### KENTUCKY STATE DENTAL SOCIETY.

The annual meeting of this society was held in the city of Louisville, on the 2d., and 3d., of June. About the usual number of members were in attendance. The meeting in many respects was a good one, especialy so far as the discussion of practical subjects was concerned.

There was quite a spirited discussion upon the relative merits of cohesive and non-cohessive gold foil for filling teeth; the chief advantages of each were pretty fully presented, and we doubt not the advocates of each received some new ideas.

There seems to be in the minds of some, who have uniformly adhered to the use of soft or non-cohesive to condemn strongly the use of co-hesive foil, some affirming that the latter is not fit to be used under any circumstances for filling

### CONTENTS.

COMMUNICATIONS:

### THE DENTAL REGISTER,

### A Monthly Magazine devoted entirely to the Interest of

### THE DENTAL PROFESSION.

**Terms Reduced to $2 50 per Year. Single Numbers 25cts.**

**EDITED BY PROF. J. TAFT,**

**And Published by SPENCER, CROCKER & CO., Ohio Dental Depot.**

The volume commences in January and closes in December.

The REGISTER being issued monthly is always fresh, therefore indispensable to the practitioner who desires to keep posted up with the new inventions and improvements which are daily developed. Neither expense nor effort will be spared to give value and variety to its contents. Articles will be illustrated with well executed engravings whenever they will add to the interest or assist in explanation.

Its extensive circulation and frequency of issue make it one of the BEST DENTAL ADVERTISING MEDIUMS in the United States.

All subscriptions must begin with January or July.

**RATES OF ADVERTISING.**

| | 1 Mo. | 3 Mo. | 6 Mo. | 1 Year. | | 1 Year. | |
|---|---|---|---|---|---|---|---|
| 1 page.... | $12 00 | $25 00 | $40 00 | $75 00 | Inserted pages must | 1st page. | $50 00 |
| ½ page ... | 8 00 | 15 00 | 25 00 | 40 00 | be plain paper and | 2d page. | 37 50 |
| ¼ page ... | 5 00 | 10 00 | 15 00 | 25 00 | black ink. | 3d page. | 33 33 |
| | | | | | | 4th page. | 25 00 |

Local or special notices, five lines or less, will be inserted for $3 each insertion: over five lines, 50 cts. per line

All advertising bills payable in advance, or at furthest, after the issue of the first number containing the advertisement. All transient advertisements to be paid for in advance.

☞ CONTRIBUTIONS SOLICITED.

Exclusively Editorial Communications should be addressed to

**DR. J. TAFT, EDITOR.**

The latest number of the REGISTER may be ordered with or without other goods at any time, and all letters should be addressed to

**SPENCER, CROCKER & CO., OHIO DENTAL DEPOT,**
Cincinnati, Ohio.

